# GC^2^MFND: Multi-Granularity Conflict and Domain-Guided Calibration for Multimodal Fake News Detection

**DOI:** 10.3390/e28060672

**Published:** 2026-06-11

**Authors:** Yanming Sun, Mingyue Zhang, Fujun Zhang

**Affiliations:** 1 School of Transportation, Shandong University of Science and Technology, Qingdao 266590, China; 2 School of Computer Science and Engineering, Shandong University of Science and Technology, Qingdao 266590, China; mingyue@sdust.edu.cn; 3 Library, Shandong University of Science and Technology, Qingdao 266590, China; libzhang@sdust.edu.cn

**Keywords:** fake news detection, multimodal learning, multi-domain learning

## Abstract

On current social media platforms, multimodal fake news has permeated various fields. Multi-domain fake news detection has garnered significant attention in the academic community. Existing multi-domain methods primarily employ feature fusion techniques based on text–image alignment, neglecting the extraction of conflicting information across modalities and failing to address the domain-dependent nature of cross-modal feature conflicts. To address this, we propose a Multi-**G**ranularity **C**onflict and Domain-**G**uided **C**alibration for **M**ultimodal Fake **N**ews **D**etection model (GC^2^MFND). This model captures conflicting features through the domain-aware multi-granularity conflict extraction module and mitigates feature suppression using the domain-guided multimodal feature calibration module. Finally, it combines domain-adaptive aggregation with multi-view evidence integration to achieve robust decision-making under supervised contrastive learning constraints. Under known domain conditions, the experimental results demonstrate that GC^2^MFND outperforms existing multi-domain baseline methods, achieving accuracy rates of 95.3%, 95.7%, and 81.2% on the Weibo, Weibo21, and FineFake datasets, respectively, representing improvements of 1.1%, 1.2%, and 1.4% over the corresponding multi-domain baselines.

## 1. Introduction

The evolution of social media has expanded the pool of news publishers from professional media organizations to individual users [1]. While this has enriched the diversity and personalization of information dissemination, it has also accelerated the creation and spread of fake news [2]. Today, news content is evolving from single-text formats to multimodal forms that integrate text, images, video, and audio [3,4,5], which has increased the inflammatory and deceptive nature of fake news [6,7,8]. The proliferation of multimodal fake news on social media easily misleads public opinion, erodes social trust, and triggers multiple social crises [9]. Meanwhile, such fake news has already permeated numerous fields [10]. From an information-theoretic perspective, the influx of multi-domain multimodal data has significantly increased the information content and diversity of news. However, this also amplifies the inherent uncertainty, complexity, and information redundancy in the fake news detection task. Traditional manual verification methods struggle to provide efficient and accurate responses in the face of massive multi-domain multimodal news. Consequently, Multimodal Fake News Detection (MFND) in different domains has attracted widespread attention [11,12,13].

**Figure 1:**
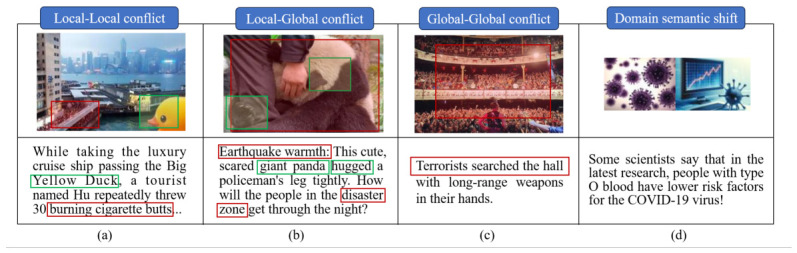
Examples of cross-modal conflict patterns and semantic shifts across different domains from the Weibo21 dataset, where the red boxes indicate conflicting semantics, while the green boxes indicate consistent semantics. (**a**) Local-Local conflict. (**b**) Local-Global conflict. (**c**) Global-Global conflict. (**d**) Domain semantic shift.

Multimodal multi-domain fake news detection methods primarily enhance detection performance by incorporating domain-specific information as auxiliary signals to learn both domain-general and domain-specific knowledge. For example, MMDFND [14] models domain-specific commonalities and peculiarities through an improved Domain Progressive Layered Extraction (DPLE) module, thereby further improving the performance of multimodal, multi-domain fake news detection. DAMMFND [15] accurately extracts domain information through domain decoupling and integrates it into a multi-view decision-making process to quantify the contributions of different modalities in detection. Multi-domain methods have optimized the incorporation of domain information into multi-view decision-making through mechanisms such as feature fusion and expert routing. However, they have overlooked semantic conflicts between features across modalities. Consequently, some studies have begun to focus on inter-modal conflicting features to enhance detection performance. Specifically, methods commonly employ co-attention, similarity, and anti-attention mechanisms to extract conflicting features. Furthermore, MIAN [16] and RaCMC [17] have validated the effectiveness of conflicting features for fake news detection. Although the aforementioned methods have achieved solid performance in multimodal fake news detection, two major issues remain:

(1) Domain dependence of cross-modal feature conflicts: In text–image conflict scenarios, the conflict pattern is not uniform across domains [18] but exhibits significant domain heterogeneity. Figure 1a shows news in the social domain, characterized by local text–image conflicts. In contrast, Figure 1b shows news in the disaster domain, characterized by conflicts between local text features and global image features. Figure 1c illustrates a global conflict at the scene level. Existing methods use a static modeling paradigm, making it hard to adaptively extract conflict features based on domain characteristics, thus insufficiently capturing latent contradictions in specific scenarios.
(2) Semantic shifts in cross-domain feature distributions: The semantic distributions of the same modality shift significantly across different domains [19,20], causing a decline in feature discriminability. The same vocabulary and image representations can have fundamentally different discriminative power across domains. As shown in Figure 1d, the keyword “virus” refers to biological pathogens in the medical domain but malicious code in the technology domain. A unified modeling approach that ignores such semantic drift loses domain-specific discriminative information, thus limiting the model’s cross-scenario generalization.

To address these challenges, we propose a Multi-**G**ranularity **C**onflict and Domain-**G**uided **C**alibration for **M**ultimodal **F**ake **N**ews **D**etection model (GC^2^MFND). Current models focus on enhancing complementary information between text and images. In contrast, GC^2^MFND treats domain embeddings as the hub of adaptive regulation and focuses on addressing the problem of underutilized modal conflicts. First, the model uses a domain-aware multi-granularity conflict extraction module. By dynamically adjusting the perception weights of local and global perspectives using domain embeddings, this module captures domain-specific conflict signals effectively. To address the issue of semantic drift, we have developed a domain-guided multimodal feature calibration module. By employing intra-modal adaptive calibration and domain-guided gated redundancy removal, we effectively reduce noise while achieving domain-adaptive semantic alignment. The model employs domain-adaptive aggregation, dynamically assigning optimal aggregation weights to conflict features and modalities based on domain characteristics to produce domain-adaptive conflict features and multimodal features. Finally, the model uses a multi-view evidence integration strategy. It fuses calibrated unimodal representations, multi-granularity conflict representations, and global semantics. This enables collaborative decision-making for complex evidence chains under domain-supervised contrastive constraints. Through this approach, GC^2^MFND mitigates detection biases caused by domain heterogeneity and enhances the utilization of inter-modal conflict features.

The main contributions in this study are summarized as follows:

(1) We propose the GC^2^MFND model that dynamically extracts modal features based on domain embeddings, integrates conflicting features with enhanced cross-modal representations, and aggregates evidence from multiple perspectives to verify the authenticity of news.
(2) We propose a domain-aware, multi-granularity conflict extraction mechanism to capture cross-modal inconsistencies at three levels: local–local, local–global, and global–global. Additionally, we achieve dynamic feature integration through a domain-adaptive aggregation framework.
(3) We construct a domain-guided feature calibration module to obtain domain-corrected features, employing multi-view evidence integration and domain contrastive learning constraints to form a complete collaborative reasoning evidence chain.

## 2. Related Works

With the continuous development of the field of news detection [21], related studies have progressively transitioned from early unimodal news detection to multimodal approaches, and with the increasing segmentation of news domains, multi-domain fake news detection has emerged as a focal point of investigation.

### 2.1. Multimodal Fake News Detection

Multimodal fake news detection generally aims to reduce the semantic gap between text and images, with a primary focus on feature fusion and network architecture design. Early studies [22] treated visual information as a supplement to textual content. For instance, EANN [23] employed a generative adversarial network to map bimodal features into a unified space for simple concatenation. Subsequently, a number of methods [24,25] adopted pretrained models for feature extraction and performed early multimodal fusion through concatenation or vector operations. Considering the higher-level semantic relationships between images and text, SpotFake [26] used a pre-trained model to extract features from images and text, and classified fake news by concatenating these features. Later, Masked Autoencoder (MAE) [27] was proposed based on masked autoencoders to improve local feature extraction, capturing subtle local manipulation traces and micro-level semantic anomalies in images more sensitively than conventional CNNs, thereby providing visual support for fine-grained conflict mining. Radford et al. [28] introduced the Contrastive Language-Image Pre-training (CLIP) model, which constructs an aligned text–image shared semantic space through large-scale contrastive learning and enhances global feature extraction. Building on this foundation, Liu et al. [29] proposed the interactive mixture-of-experts framework MIMoE-FND, which explicitly models semantic alignment degree and unimodal consistency while employing gating mechanisms for adaptive feature fusion. Since simple fusion methods struggle to identify conflicts such as “text–image irrelevance” or “text–image contradiction,” research focus has shifted toward cross-modal inconsistency mining. CAFE [30] was the first to quantify the degree of modality conflict using KL divergence and dynamically adjust fusion weights accordingly. RaCMC [17] leveraged knowledge distillation to maximize modal interaction information for detecting anomalous image–text relationships. TLFND [31] extracted text–image conflicts at multiple levels, including local and global levels as well as intra-modal and inter-modal levels, through a three-level feature matching distance mechanism. However, most existing studies adopt a uniform cross-modal interaction mechanism that does not fully account for feature variations across different news domains, thereby limiting model performance in multi-domain scenarios [32].

### 2.2. Multi-Domain Fake News Detection

Real-world news data spans numerous domains and is highly heterogeneous. Achieving domain-adaptive detection is the goal of multi-domain fake news detection [33,34,35,36]. KATMF [37] was the first to combine multi-domain and multimodal approaches for fake news detection, utilizing adversarial multi-task learning and knowledge-enhanced Transformers to capture differences in the feature distributions of news articles across different domains. EMT [38] improved generalization by extracting both domain-specific and domain-invariant features and by incorporating external knowledge. To address domain distribution shifts, Zhang et al. [39] and Li et al. [40] utilized Bidirectional Encoder Representations from Transformers (BERT) and transfer learning to mitigate cross-domain discrepancies. To model the specific distributions of different domains with greater precision, Mixture of Experts (MoE) and graph-structured learning have emerged as mainstream approaches in recent years. To address the issue of domain data imbalance, MDFEND [10] employed a MoE architecture and a domain gating mechanism to dynamically integrate expert representations. M^3^DFEND [41] and MMDFND [14] further enhanced multi-view adaptive aggregation through domain adapters and an improved DPLE module, respectively. Zhao et al. [42] utilized a mixture-of-experts network and a gating mechanism to address feature distribution discrepancies in multi-domain fake news detection. Yuan et al. [43] modeled cross-domain relationships among news events based on a graph attention network, utilizing structured information to aid in identification. Recently, Lu et al. [15] proposed DAMMFND, which further introduced the concept of feature decoupling. By separating domain features from semantic features and combining them with a domain-aware decision mechanism, it achieved a deep analysis of domain heterogeneity. Xu et al. [44] proposed DATTAMM, which employed a domain-aware test-time adaptation mechanism to dynamically adjust model parameters during the inference stage, thereby accommodating the feature distribution of the target domain. However, hard decoupling may filter out subtle counterfeit detection cues by disrupting the semantic flow of text and images. Therefore, the key to improving multi-domain multimodal fake news detection is to fully leverage cross-modal conflict information and discriminative features, guided by domain-specific knowledge, without compromising core semantic meaning.

## 3. Methods

**Figure 2:**
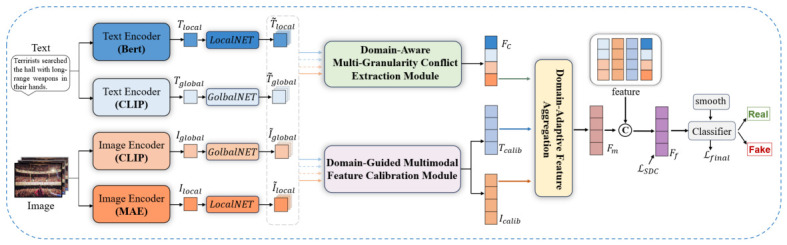
The network architecture of GC^2^MFND. BERT, MAE, and CLIP are utilized to extract multi-granularity features of multimodal news. Domain-Aware Conflict Extraction is employed to mine subtle visual–textual conflicts. Domain-Guided Feature Calibration enhances visual–textual features via domain information. Domain-Adaptive Feature Aggregation generates weights based on domain embeddings to aggregate multi-channel features. News authenticity is determined by the concatenated features.

In this section, we introduce the proposed GC^2^MFND, whose overall architecture is shown in Figure 2. Given a news sample containing text, images, and domain labels, our method first performs multi-view feature encoding and domain embedding generation (Section 3.1). Subsequently, the model comprises three core modules: a module for mining cross-modal conflicts across local-to-global scales (Section 3.2), a module for domain-guided dynamic feature calibration and deduplication (Section 3.3), and a domain-adaptive feature aggregation module (Section 3.4).

Each input multimodal news sample is represented as $N=[T,I,D_{d}]\in D$, where *T*, *I*, $D_{d}$, and D denote the text content, image content, domain label, and dataset, respectively. The news items in the entire dataset are classified into *k* domains, with each domain assigned a label $D_{d}\in \left\{D_{1},D_{2},D_{3},\ldots ,D_{k}\right\}$. The objective of multimodal domain-adaptive fake news detection is as follows: given multimodal content comprising text *T* and image *I*, and using the explicit domain label $D_{d}$ as prior knowledge, the model determines the authenticity of the news item via a domain-adaptive mechanism. The main symbols used in this method and their meanings are shown in Table 1.

### 3.1. Multi-Granularity Feature Extraction and Domain Representation Module

To exhaustively mine the discriminative features of each modality and enhance the representation capability, we employ a dual-granularity feature encoding module that simultaneously extracts fine-grained local features and coarse-grained global features from text and image modalities to capture semantic information at different levels.

#### 3.1.1. Fine-Grained Local Feature Extraction

Given a text *T*, we use the pre-trained BERT model [45] as a text encoder to obtain fine-grained local features of the text, denoted as $T_{\mathrm{local}}\in R^{L\times d_{t}}$, where *L* is the length of the text sequence and $d_{t}$ is the dimension of the text features. Meanwhile, given an image *I*, we use the MAE [27] model to extract patch features as fine-grained local image features, denoted as $I_{\mathrm{local}}\in R^{P\times d_{i}}$, where *P* is the number of image patches and $d_{i}$ is the image feature dimension. To uniformly compress and align text–image features from the high-dimensional pre-training space, we define LocalNET as a local feature adapter, employing a multi-scale one-dimensional convolutional extractor [46]. We can obtain the enhanced text local features ${\tilde{T}}_{\mathrm{local}}={\mathrm{LocalNET}}_{\mathrm{text}}\left(T_{\mathrm{local}}\right)\in R^{L\times d}$ and the enhanced local image features ${\tilde{I}}_{\mathrm{local}}={\mathrm{LocalNET}}_{\mathrm{img}}\left(I_{\mathrm{local}}\right)\in R^{P\times d}$ using the method described above.

#### 3.1.2. Coarse-Grained Global Feature Extraction

Global features are designed to provide coarse-grained, macro-level semantic information. We utilize a pre-trained CLIP model [28] to extract global features for both images and text. The text content is processed by CLIP’s text encoder to obtain global text features $T_{\mathrm{global}}\in R^{d_{g}}$ that represent the overall semantic meaning. Similarly, the image content is encoded by CLIP’s image encoder to obtain image global features $I_{\mathrm{global}}\in R^{d_{g}}$ that encapsulate high-level visual semantics, where $d_{g}$ is the dimension of the global features. Similarly, to ensure consistency with the aforementioned local features within a unified metric space, GlobalNET is defined as a global feature adapter. It employs linear projection and layer normalization to map these features into a unified high-dimensional semantic space. Thus, we obtain the enhanced text global features ${\tilde{T}}_{\mathrm{global}}={\mathrm{GlobalNET}}_{\mathrm{text}}\left(T_{\mathrm{global}}\right)\in R^{d}$ and the enhanced image global features ${\tilde{I}}_{\mathrm{global}}={\mathrm{GlobalNET}}_{\mathrm{img}}\left(I_{\mathrm{global}}\right)\in R^{d}$.

#### 3.1.3. Domain Embeddings

Given the substantial statistical heterogeneity in fabrication patterns and content distribution across various news domains, we introduce learnable domain embeddings to enable the model to capture domain-specific characteristics. Domain labels are fed into the embedding layer to produce domain embedding vectors $e_{d}=E_{\mathrm{dom}}\left[d,:\right]\in R^{d_{d}}$, where $E_{\mathrm{dom}}\in R^{N\times d_{d}}$ and *N* denotes the number of domains.

### 3.2. Domain-Aware Multi-Granularity Conflict Extraction Module

**Figure 3:**
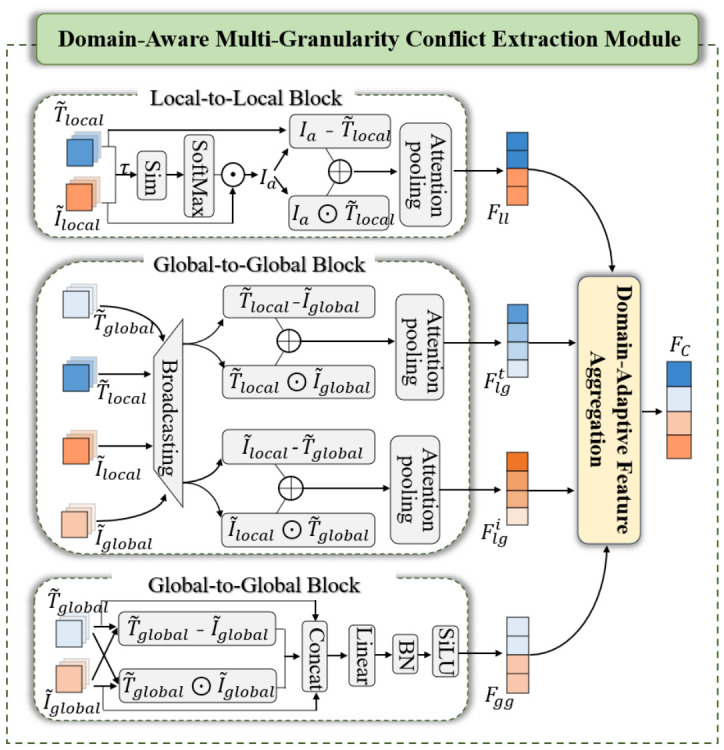
Architecture of a Domain-Aware Multi-Granularity Conflict Extraction Module.

In multimodal fake news detection, semantic conflicts between text and images serve as key clues for identifying fake news. To better capture and utilize conflict features across various domains, we propose a domain-aware, multi-granularity conflict extraction module, as shown in Figure 3. Conflict features are extracted from three perspectives: “local–local,” “local–global,” and “global–global.” The contribution of these features is adaptively adjusted under the influence of the domain embedding $e_{d}$.

To address the discrepancies in dimensionality and information density between local and global features, we design two asymmetric cross-modal interaction operators [30] to extract conflicting information across modalities of different granularities [18]. For local features, we employ a parameter-free, heuristic element-level operator $F_{seq}$ to amplify anomalous deviation signals. It is defined as:

(1) $$F_{seq}\left(X,Y\right)=\left|X-Y\right|+\left(X⊙Y\right)$$

where *X* and *Y* denote the image and text features, respectively; the absolute difference term quantifies the numerical deviation between the two features, serving to capture fine-grained semantic contradictions; and the product term measures the co-occurrence patterns of the two features in the feature space.

To address global features and to mitigate the loss of nonlinear conflict patterns that occur when traditional cosine metrics compress high-dimensional information into a single scalar, a multidimensional heuristic interactive mapping operator H is employed to extract robust macroscopic conflict representations while preserving the modal context. It is defined as:

(2) $$H\left(X,Y\right)=\mathrm{SiLU}\left(\mathrm{BN}\left(W_{h}\left[X\|Y\|(X-Y)\|(X⊙Y)\right]\right)\right)$$

Local-Local view: The local–local conflict feature aims to capture fine-grained inconsistencies between text words and local image patches. First, the local text features and local image features are $L_{2}$-normalized (i.e., Euclidean normalization), after which a similarity matrix smoothed by a learnable temperature coefficient τ is computed:

(3) $$S_{ll}=\frac{{\tilde{T}}_{local}{\tilde{I}}_{local}^{⊤}}{\tau }$$

Then, after applying a Softmax function to $S_{ll}\in R^{L\times P}$ along the image block dimension and weighting the local image features, we obtain a text-aligned image sequence $I_{a}=\mathrm{Softmax}\left(S_{ll}\right){\tilde{I}}_{local}$. Based on the aligned features, we employ the lightweight sequence conflict operator to obtain the local–local conflict sequence $C_{ll}=F_{seq}\left(I_{a},T_{local}\right)$. And through a text-mask-aware attention pooling layer, we derive the local conflict features $F_{ll}$:

(4) $$F_{ll}=u_{ll}^{⊤}C_{ll},\;u_{ll}=\mathrm{softmax}\left(W_{u}\phi \left(C_{ll}\right)\right)$$

where $W_{u}$ is a trainable parameter and ϕ(·) denotes a non-linear mapping function.

Local–Global view: Local–global conflict features capture the semantic discrepancy between fine-grained local elements and the overall global context. They address the semantic misalignment between local and global elements across different modalities. Specifically, we employ a broadcast extension mechanism. This mechanism spatially aligns local features with global features, enabling direct comparison between each local element and the corresponding cross-modal global features. Then, we apply the lightweight sequence conflict operator. This produces the “local text–global image” conflict sequence $C_{lg}^{t}=F_{seq}\left({\tilde{T}}_{local},{\tilde{I}}_{global}\right)$ and the “local image–global text” conflict sequence $C_{lg}^{i}=F_{seq}\left({\tilde{I}}_{local},{\tilde{T}}_{global}\right)$. Using the attention pooling layer, we obtain the local text–global image conflict features $F_{lg}^{t}$ and the local image–global text conflict features $F_{lg}^{i}$:

(5) $$F_{lg}^{t}={\left(u_{lg}^{t}\right)}^{⊤}C_{lg}^{t},\;u_{lg}^{t}=\mathrm{softmax}\left(\phi \left(C_{lg}^{t}\right)W_{lg}^{t}\right)$$

(6) $$F_{lg}^{i}={\left(u_{lg}^{i}\right)}^{⊤}C_{lg}^{i},\;u_{lg}^{i}=\mathrm{softmax}\left(\phi \left(C_{lg}^{i}\right)W_{lg}^{i}\right)$$

Global–Global view: Global–Global conflict features capture the overall semantic inconsistency between the text and the image. To mitigate the loss of multidimensional contradictory information and complex nonlinear patterns, we employ the interactive mapping operator to extract a high-dimensional global conflict representation $F_{gg}=H\left({\tilde{T}}_{\mathrm{global}},{\tilde{I}}_{\mathrm{global}}\right)$. This approach helps preserve a rich representation of global cross-modal conflict features while preserving the topological structure of the high-dimensional space.

Since multi-granularity conflict feature patterns vary across different domains, we leverage the domain-adaptive feature aggregation module described later to achieve domain-adaptive fusion of these conflict features. Through this module, we generate weight vectors $w_{ll},w_{lg}^{t},w_{lg}^{i},w_{gg}\in R^{d}$ that represent the importance of $\mathrm{LL},{\mathrm{LG}}^{t},{\mathrm{LG}}^{i}\;\mathrm{and}\;\mathrm{GG}$ conflicts in the current domain, thereby obtaining the domain-aware conflict feature $F_{C}$:

(7) $$F_{C}=w_{ll}⊙F_{ll}+w_{lg}^{t}⊙F_{lg}^{t}+w_{lg}^{i}⊙F_{lg}^{i}+w_{gg}⊙F_{gg}$$

### 3.3. Domain-Guided Multimodal Feature Calibration Module

**Figure 4:**
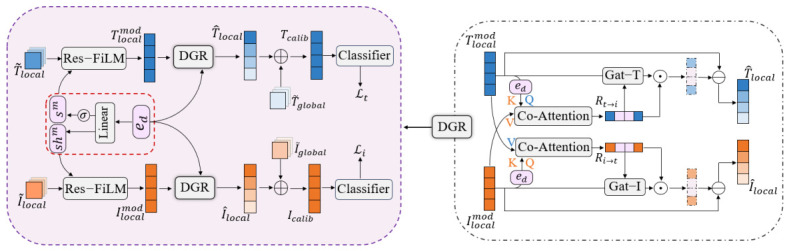
Architecture of the domain-guided multimodal feature calibration module.

To mitigate semantic distribution discrepancies across domains and enhance the domain adaptability of features, we propose a domain-guided feature calibration module, whose overall structure is illustrated in Figure 4. This module takes domain embeddings $e_{d}$ as prior conditioning information and conducts stepwise calibration and enhancement of local text and image features via three steps: Conditional Modulation, Domain-guided Gated Redundancy Removal (DGR), and Global Semantic Compensation.

To achieve domain-aware feature calibration and preserve general semantic information during cross-domain alignment, we introduce a residual-based linear modulation mechanism, termed Res-FiLM. In contrast to direct fusion of domain labels, we use the domain embedding $e_{d}$ as a prior condition to generate, via independent affine transformations, dynamic scaling factors $s^{m}$ and offsets ${\mathrm{sh}}^{m}$ for the current input sample. We then use these generated parameters to adaptively calibrate the local text features ${\tilde{T}}_{\mathrm{local}}$ and local image features ${\tilde{I}}_{\mathrm{local}}$, yielding the modulated features $T_{\mathrm{local}}^{\mathrm{mod}}$ and $I_{\mathrm{local}}^{\mathrm{mod}}$. The formula is as follows:

(8) $$s^{m}=\sigma \;\left(W_{\mathrm{scale}}^{m}e_{d}\right)\in R^{d},\;sh^{m}=W_{\mathrm{shift}}^{m}e_{d}\in R^{d}$$

(9) $$T_{\mathrm{local}}^{\mathrm{mod}}={\tilde{T}}_{\mathrm{local}}+\left({\tilde{T}}_{\mathrm{local}}⊙s^{t}+sh^{t}\right),\;I_{\mathrm{local}}^{\mathrm{mod}}={\tilde{I}}_{\mathrm{local}}+\left({\tilde{I}}_{\mathrm{local}}⊙s^{i}+sh^{i}\right)$$

where σ(·) represents the sigmoid activation function; m∈{t,i} denotes the text (*t*) or image (*i*) modality; $W_{\mathrm{scale}}^{*}$ and $W_{\mathrm{shift}}^{*}$ are learnable projection matrices for the corresponding modalities; and ⊙ denotes element-wise multiplication.

Since the definition of “redundancy” varies between domains, we employ DGR to enhance the discriminative power of text–image features. To obtain cross-modal redundant representations, we first employ the domain embedding $e_{d}$ to dynamically modulate the query and key matrices:

(10) $$D_{Q}=\sigma \left(W_{q}^{D}e_{d}\right),\;D_{K}=\sigma \left(W_{k}^{D}e_{d}\right)$$

Taking text features as an example, we use the modulated matrix to compute the domain-guided cross-modal co-occurrence attention matrix $A_{t\to i}$, which is then aggregated to form the text redundancy representation $R_{t\to i}$:

(11) $$A_{t\to i}=\mathrm{Softmax}\left(\frac{\left(T_{\mathrm{local}}^{\mathrm{mod}}⊙D_{Q}\right){\left(I_{\mathrm{local}}^{\mathrm{mod}}⊙D_{K}\right)}^{⊤}}{\sqrt{d}}\right)$$

(12) $$R_{t\to i}=A_{t\to i}I_{\mathrm{local}}^{\mathrm{mod}}$$

Similarly, we compute the image attention matrix $A_{i\to t}$ and extract the image-to-text redundant representation $R_{i\to t}$.

Next, we employ nonlinear adaptive subtractive gating to obtain clean features. An adaptive gating function $g_{t}$ is generated via a deep neural network to filter out redundant information, which is then subtracted from the modulated features to obtain the purified local text features ${\hat{T}}_{\mathrm{local}}$:

(13) $$g_{t}=\sigma \left(W_{gt}\left[T_{\mathrm{local}}^{\mathrm{mod}},R_{t\to i}\right]\right),\;{\hat{T}}_{\mathrm{local}}=T_{\mathrm{local}}^{\mathrm{mod}}-\left(g_{t}⊙R_{t\to i}\right)$$

Similarly, we can obtain the local features of the clean image ${\hat{I}}_{\mathrm{local}}$.

Although subtraction-based decoupling highlights micro-level cues, excessive orthogonalization may weaken macro-level text coherence and global contextual image dependencies. To compensate for this potential loss of semantic information, we introduce global anchor features ${\tilde{T}}_{\mathrm{global}}$ and ${\tilde{I}}_{\mathrm{global}}$ to restore semantic integrity. This yields domain-adaptive and discriminative calibrated features: $T_{\mathrm{calib}}$ for text and $I_{\mathrm{calib}}$ for images, which serve as unimodal features for the subsequent module. A mask is applied to the text features to remove placeholders, as shown in the following formula:

(14) $$T_{calib}=\mathrm{SiLU}\left(\mathrm{BN}\left(W_{out}^{t}F_{attn}\left({\hat{T}}_{local},Mask\right)⊕{\tilde{T}}_{global}\right)\right)$$

(15) $$I_{calib}=\mathrm{SiLU}\left(\mathrm{BN}\left(W_{out}^{i}F_{attn}\left({\hat{I}}_{local}\right)⊕{\tilde{I}}_{global}\right)\right)$$

### 3.4. Domain-Adaptive Feature Aggregation Module

In this module, we design a dynamic weight generation network to achieve domain-adaptive feature fusion. Given a domain embedding vector $e_{d}$, the gated network learns the mapping relationship between domain attributes and feature discriminative power through non-linear projection. Specifically, for each feature branch v∈{1,2,…,n}, it dynamically generates a corresponding feature-wise weight vector $w_{v}\in R^{d}$:

(16) $$w_{v}=\sigma \left({\mathrm{Linear}}_{v}\left(e_{d}\right)\right)$$

(17) $$F_{j}=w_{1}⊙F_{1}+w_{2}⊙F_{2}+\cdots +w_{n}⊙F_{n}$$

This module is reused twice in the model. The first application occurs during the multi-granularity conflict extraction stage, where the previously described method generates weights to dynamically fuse the most domain-representative conflicting signals, thereby obtaining the conflict feature $F_{C}$. The second reuse occurs during the adaptive aggregation of integrated features. In this stage, the calibrated text features $T_{\mathrm{calib}}$, image features $I_{\mathrm{calib}}$, and conflict features $F_{C}$ from the preceding module are fused in a domain-adaptive manner. This process similarly generates corresponding weight vectors $w_{t},w_{i},w_{c}\in R^{d}$ to obtain information-rich fused multimodal features $F_{m}$.

(18) $$F_{m}=w_{t}⊙T_{calib}+w_{i}⊙I_{calib}+w_{c}⊙F_{C}$$

### 3.5. Multi-View Evidence Integration and Loss Functions

After obtaining refined text features $T_{\mathrm{calib}}$, image features $I_{\mathrm{calib}}$, multi-granularity conflict features $F_{C}$, and fused modal features $F_{m}$ from the above modules, we adopt an evidence-based strategy that concatenates all these features and feeds them into a deep fusion network. The network generates the final classification features $F_{f}$, which are then fed into the classifier.

(19) $$F_{f}=\mathrm{MLP}\left(\mathrm{concat}\left(T_{calib},I_{calib},{\tilde{T}}_{global},{\tilde{I}}_{global},F_{C},F_{m}\right)\right)$$

In multi-domain joint training, fake news exhibits semantic heterogeneity across different domains. Traditional supervised contrastive learning [47], which does not distinguish domain boundaries, tends to push all samples with the same label to cluster tightly together in the feature space. To mitigate feature interference in different domains, we introduce a domain-aware soft-weighted contrastive loss $L_{\mathrm{DSC}}$ to bring samples of the same class within the same domain closer to each other to varying degrees, while pushing samples of different classes further apart. We define domain-aware positive sample masks $M_{i,j}^{\mathrm{pos}}$ and negative sample masks $M_{i,j}^{\mathrm{neg}}$ as follows:

(20) $$M_{i,j}^{\mathrm{pos}}=\left\{\begin{array}{cc} 1, & y_{i}=y_{j},D_{i}=D_{j},i\neq j\\ \theta , & y_{i}=y_{j},D_{i}\neq D_{j},i\neq j\\ 0, & \mathrm{otherwise} \end{array}\right.\;\mathrm{and}\;M_{i,j}^{\mathrm{neg}}=\left\{\begin{array}{cc} 1, & y_{i}\neq y_{j}\\ 0, & \mathrm{otherwise} \end{array}\right.$$

(21) $$L_{\mathrm{DSC}}=\frac{-1}{W_{\mathrm{pos}}}\sum_{i=1}^{N}\sum_{j=1}^{N}M_{i,j}^{\mathrm{pos}}\log\frac{\exp\left(F_{i}\cdot F_{j}/\tau \right)}{\sum_{k=1}^{N}M_{i,k}^{\mathrm{all}}\exp\left(F_{i}\cdot F_{k}/\tau \right)}$$

where τ denotes the temperature coefficient, θ∈(0,1) is a hyperparameter controlling the strength of cross-domain positive sample alignment, $W_{\mathrm{pos}}=\sum_{i=1}^{N}\sum_{j=1}^{N}M_{i,j}^{\mathrm{pos}}$, $M_{i,j}^{\mathrm{all}}=M_{i,j}^{\mathrm{pos}}+M_{i,j}^{\mathrm{neg}}$, and $F_{i}$ and $F_{j}$ are the final classification features.

Since all global classification and auxiliary supervision tasks are essentially binary classification problems, and to mitigate the risk of overfitting caused by overconfidence in deep neural networks, all classifiers in this model uniformly adopt the binary cross-entropy loss with a label smoothing strategy, ${\mathrm{BCE}}_{\mathrm{smooth}}\left(\hat{y},y\right)$.

(22) $${\mathrm{BCE}}_{\mathrm{smooth}}\left(\hat{y},y\right)=-\frac{1}{B}\sum_{i=1}^{B}\left[{\tilde{y}}_{i}\log\left({\hat{y}}_{i}\right)+\left(1-{\tilde{y}}_{i}\right)\log\left(1-{\hat{y}}_{i}\right)\right]$$

where *B* denotes the given batch size, ${\hat{y}}_{i}$ represents the predicted probability, $y_{i}\in \left\{0,1\right\}$ denotes the original true label of the sample, and ${\tilde{y}}_{i}=y_{i}\left(1-\epsilon \right)+\frac{\epsilon }{2}$ denotes the smoothed true label obtained by introducing the smoothing parameter ϵ.

To enhance the discriminative power of each modal feature in fake news detection, we apply independent classification supervision to the conflict features, text features, image features and the final fused features, calculating their respective losses as $L_{\mathrm{conflict}}$, $L_{t}$, $L_{i}$ and $L_{\mathrm{final}}$. Consequently, the total loss for GC^2^MFND is as follows:

(23) $$L_{\mathrm{total}}=L_{\mathrm{final}}+\alpha \cdot L_{\mathrm{conflict}}+\beta \cdot \frac{L_{t}+L_{i}}{2}+\gamma \cdot L_{\mathrm{DSC}}$$

where α, β, and γ are weight coefficients for balancing the losses of different terms.

## 4. Experiments

In this section, we conduct an empirical evaluation of GC^2^MFND using three datasets covering news from different domains. The experiments in this section aim to elucidate the six dimensions of interest in this study concerning fake news detection by exploring the following research questions:

**RQ1.**  Does GC^2^MFND effectively improve the overall performance of fake news detection?
**RQ2.**  Can GC^2^MFND improve the detection accuracy for specific types of fake news?
**RQ3.**  Does each component of GC^2^MFND contribute to improved detection?
**RQ4.**  Is the domain-adaptive fusion mechanism capable of effectively capturing feature distribution discrepancies across different domains?
**RQ5.**  How sensitive is GC^2^MFND to key hyperparameters, and what is its parameter robustness?
**RQ6.**  Does GC^2^MFND exhibit high computational efficiency during both the training and inference stages?

### 4.1. Experimental Settings

#### 4.1.1. Datasets

We evaluate GC^2^MFND on three real-world datasets: Weibo [23], Weibo21 [10], and FineFake [48]. For the Weibo dataset, we adopt the same data splitting and domain classification methods as the baseline work [14], dividing the data into training, validation, and test sets at a ratio of 7:1:2, and categorizing it into nine domains: finance, healthcare, military, science, politics, disasters, education, entertainment, and society. Weibo21 is a larger, multi-domain multimodal dataset covering data up to 2021. Following the partitioning scheme of the benchmark method [14], we split it into training, validation, and test sets in an 8:1:1 ratio. This dataset is categorized into nine domains: finance, health, military, science, politics, international affairs, education, entertainment, and society. Both of the above datasets are Chinese datasets sourced from the Weibo news platform. FineFake is a larger, multi-domain multimodal fake news detection dataset covering data up to 2024. It is split into training, validation, and test sets in a 6:2:2 ratio and includes data from eight news platforms, such as Twitter and Snopes, covering six domains: politics, entertainment, business, health, society, and conflict. The domain labels for each dataset were manually annotated. Furthermore, to ensure data quality, we follow the preprocessing steps outlined in previous works [14,48,49,50] to prevent data leakage between the training and test sets. To ensure a fair comparison, we obtain the experimental results of all baselines using the same dataset partitioning and pre-processing methods described above. See Table 2 for the data volume.

#### 4.1.2. Implementation Details

In the multi-granularity feature extraction phase, BERT, MAE, and CLIP are used to extract image–text features. The parameters of their backbone networks are frozen, and corresponding Chinese and English BERT and CLIP models are used to accurately extract multilingual features. In image–text feature extraction, the pixels of the input images are uniformly resized to 224×224, the length of local image–text features is set to 197, and the dimensionality is 768. Feature matching is performed using feature adapters, which employ parallel 1D convolutions (with kernel sizes of 1, 3, and 5) and the SiLU activation function to align the dimensions of local and global features in the image–text data to 320. The domain embedding dimension is 128. In the attention masking mechanism, the weights of invalid positions are set to the minimum value. In the loss function section, we use a binary cross-entropy loss function with label smoothing ϵ=0.1 for all classifiers, and a positive sample mask θ=0.5 in $L_{\mathrm{DSC}}$. The hyperparameters for the overall joint loss function are configured according to the differences between the Chinese and English datasets. For the two Chinese datasets, we set α=0.3, β=0.2, γ=0.7, and τ=0.1. For the English dataset FineFake, we set α=0.1, β=0.1, γ=0.05, and τ=0.1. For model optimization, we employ Adam [51] for end-to-end parameter updates, with an initial learning rate of $1\times 10^{-4}$. To prevent gradient explosion, gradient clipping is set to 1.0, the maximum number of epochs is set to 50, and early stopping is applied. All code is executed on an NVIDIA GeForce RTX 3090 graphics processing unit.

#### 4.1.3. Baseline

To conduct a comprehensive evaluation of this model, we compare it with unimodal multi-domain, multimodal multi-domain and multimodal single-domain fake news detection methods.

(1) Unimodal Multi-Domain
  - **MOSE** [52], which employs Long Short-Term Memory (LSTM) networks as the expert components in the MMoE architecture.
  - **KATMF** [37], using adversarial multi-task learning and an external knowledge base enhanced Transformer to capture feature differences in multi-domain multimodal news.
  - **MDFEND** [10], which employs a domain gate to aggregate MoE experts in a weighted manner for multi-domain fake news detection.
(2) Multimodal Multi-Domain
  - **M^3^DFEND** [43], which adaptively aggregates semantic, sentiment, and stylistic features via domain adapters and a domain memory bank.
  - **MMDFND** [14], which uses Improved PLE to capture cross-domain and specific knowledge for multi-domain multimodal fake news detection.
  - **DAMMFND** [15], which employs domain decoupling to separate domains from semantic features, and uses a domain-aware, multi-view discriminator along with a decision layer to dynamically weigh multimodal information.
(3) Multimodal Single-Domain
  - **EANN** [23], which employs a Generative Adversarial Network (GAN) to learn event-invariant general knowledge.
  - **SpotFake** [26], which leverages VGG for image feature extraction and BERT for text feature extraction in fake news detection.
  - **CAFE** [30], which employs cross-modal ambiguity for the adaptive aggregation of unimodal features and cross-modal correlations.
  - **BMR** [53], which fuses multi-view features with cross-modal consistency using a weighted scheme.
  - **MIAN** [16], which extracts intra-modal and inter-modal conflict features via a reverse attention mechanism.
  - **MTS** [54], which explicitly captures multi-order text–image interactions via Taylor series expansion, reduces model parameters and increases interpretability.

### 4.2. Overall Performance

To address RQ1 and RQ2, this section presents comparative experiments between GC^2^MFND and the three representative baselines described above, and analyzes the experimental results in terms of both overall performance and F1 scores for various domains. For the Weibo and Weibo21 datasets, the existing state-of-the-art results were taken from prior experiments [14] and are marked with an asterisk (*) in Table 3. For the newly introduced FineFake dataset, given the limited publicly available experimental results for existing methods, we reproduce the results for each baseline method under a standardized experimental setup and reported these findings.

As shown in Table 3, GC^2^MFND is compared with representative state-of-the-art multi-domain fake news detection baseline methods across three benchmark datasets and achieves the best results in terms of overall evaluation metrics. On the Weibo dataset, GC^2^MFND achieves overall F1, Acc, and AUC scores of 0.953, 0.953, and 0.986, respectively, representing improvements of 1.1%, 1.1%, and 0.4% over the best competing method. On the Weibo21 dataset, GC^2^MFND achieves overall F1, Acc, and AUC scores of 0.957, 0.957, and 0.986, respectively, representing improvements of 1.2%, 1.2%, and 0.3% over the best competing method. On the larger and more diverse FineFake dataset, GC^2^MFND achieves overall F1, Acc, and AUC scores of 0.807, 0.812, and 0.890, respectively, representing improvements of 1.2%, 1.3%, and 0.8% over the best baseline method.

GC^2^MFND remains highly competitive in terms of F1 scores for most domains. On the Weibo dataset, GC^2^MFND achieves the best results in the military, education, society, political, and health domains, and ties with DAMMFND in the science domain. However, DAMMFND performs better in the finance, entertainment, and disaster domains. On the Weibo21 dataset, GC^2^MFND achieves the best results in the science, military, education, politics, finance, entertainment, and international domains, but performs slightly worse than some comparison methods in the society and health domains. On the FineFake dataset, GC^2^MFND achieves the best results in the society, political, health, and finance domains, but performs slightly worse in the entertainment and conflict domains. We attribute the performance differences in different domains primarily to imbalanced sample distributions and domain-specific heterogeneity. On one hand, domains with larger sample sizes benefit from stronger supervisory signals, while resource-poor domains are more prone to training biases, causing fluctuations in detection performance among different domains. On the other hand, differences in topic attributes, semantic expressions, and text–image association patterns among different domains increase detection difficulty. In particular, the FineFake dataset introduces cross-platform heterogeneity, which leads to more pronounced performance fluctuations and a significantly lower overall detection performance compared to the two Chinese datasets. Nevertheless, GC^2^MFND still outperforms baselines in most domains and enhances overall detection performance by mitigating domain heterogeneity.

To validate the stability of our method, we repeat the experiments under ten different random seeds, compute the mean and standard deviation of GC^2^MFND and two strong baselines, and then confirm the statistical significance of the performance improvements over the strong baselines using a *t*-test (*p* < 0.05), as shown in Table 4.

Table 5 presents the comparison results between GC^2^MFND and multimodal single-domain detection methods, including accuracy and F1 scores for fake and real news. Overall, GC^2^MFND achieves the best performance on all three datasets. Specifically, in terms of overall accuracy, GC^2^MFND outperforms the best baseline methods by 1.7%, 1.9%, and 2.4% on the Weibo, Weibo21, and FineFake datasets, respectively. For the fake news F1 score, the improvements are 1.9%, 2.0%, and 1.8% for the respective datasets; for the real news F1 score, the improvements are 1.5%, 1.8%, and 2.7%. These results indicate that GC^2^MFND not only outperforms single-domain multimodal detection methods in overall classification performance but also exhibits enhanced recognition capabilities for both fake and real news samples. It can be observed that the Chinese datasets show a greater improvement for fake news, whereas the English dataset shows a greater improvement for real news. This improvement primarily stems from GC^2^MFND’s ability to effectively extract multi-granularity conflict features. In Chinese datasets, where conflicts in fake news are prominent, the model achieves high accuracy. Meanwhile, particularly in the English dataset, the accompanying images, intended to enrich multimodal news presentation, cause even real news to exhibit minor conflicts. By leveraging this rich conflict information, the model distinguishes between real and fake news, thereby reducing false positives for real news.

**Figure 5:**
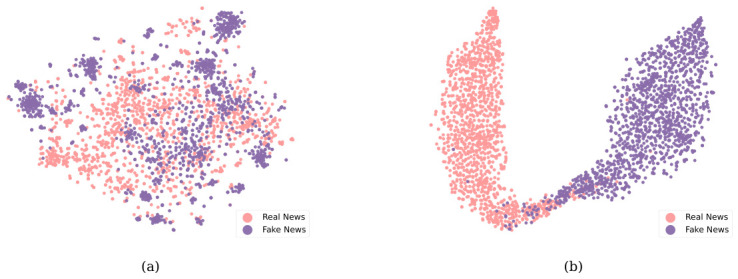
T-SNE of sample distribution on Weibo, where (**a**) shows the distribution of original samples, and (**b**) shows the distribution of learned features.

**Figure 6:**
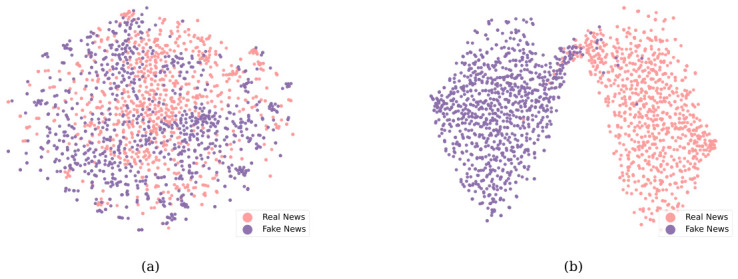
T-SNE of sample distribution on Weibo21, where (**a**) shows the distribution of original samples, and (**b**) shows the distribution of learned features.

**Figure 7:**
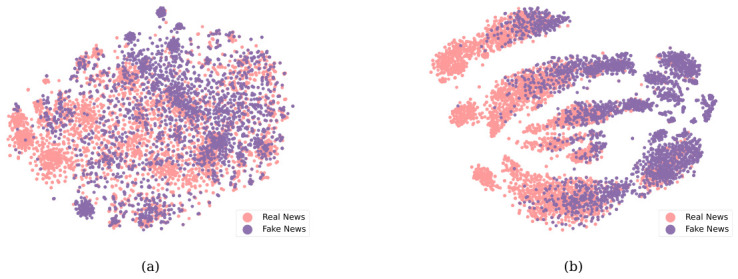
T-SNE of sample distribution on FineFake, where (**a**) shows the distribution of original samples, and (**b**) shows the distribution of learned features.

Figure 5, Figure 6 and Figure 7 show the t-SNE visualizations of the sample distributions produced by the model on the Weibo, Weibo21, and FineFake datasets. Parameters are set as follows: perplexity = 40, PCA initialization, and random seed = 3074, consistent with the baseline experiments. In Figure 5a, Figure 6a and Figure 7a, real and fake news samples are intermingled, whereas in Figure 5b, Figure 6b and Figure 7b, real and fake news exhibit relatively good separability, with only a few samples not fully separated. This demonstrates the effectiveness of GC^2^MFND in multimodal fake news classification.

### 4.3. Ablation Study

To assess the impacts of key components of GC^2^MFND on detection performance, we construct the following model variants: (1) -w/o Conflict: removal of the domain-aware multi-granularity conflict extraction module; (2) -w/o Calib: removal of the domain-guided multimodal feature calibration module; (3) -w/o Domain: removal of the domain embedding-based feature processing component; (4) -w/o Loss: removal of the contrastive loss and auxiliary loss; and (5) -w/o Smooth: removal of the label smoothing strategy during training.

Table 6 shows the experimental results. We use accuracy and F1 score to quantify the contribution of each module. Specifically, we summarize the following points:

- Comparing the first three variants, we observe that GC^2^MFND -w/o Conflict, GC^2^MFND -w/o Calib, and GC^2^MFND -w/o Domain all exhibit a performance drop, suggesting that extracting conflict features, calibrating text–image features, and incorporating domain information contribute to the performance enhancement of our model. Notably, completely removing the domain labels causes a slight performance drop of about 1%, but the model still retains high detection accuracy, showing reasonable robustness to missing domain labels. From an information-theoretic perspective, conflict features enhance the correlation between news content and truth labels, modal calibration reduces redundancy entropy among features, and domain embedding lowers conditional entropy across topics; together, these three factors improve the model’s discriminative ability in uncertain environments.
- Comparing GC^2^MFND -w/o Loss with GC^2^MFND -w/o Smooth shows that effective training and learning enhance model performance. Removing either component degrades model performance. This indicates that the contrastive loss and auxiliary loss enhance the discriminative power of features, while label smoothing prevents the model from over-relying on training samples and thus improves classification stability.

### 4.4. Discussions

#### 4.4.1. Evaluation of Domain-Adaptive Fusion Mechanisms

**Figure 8:**
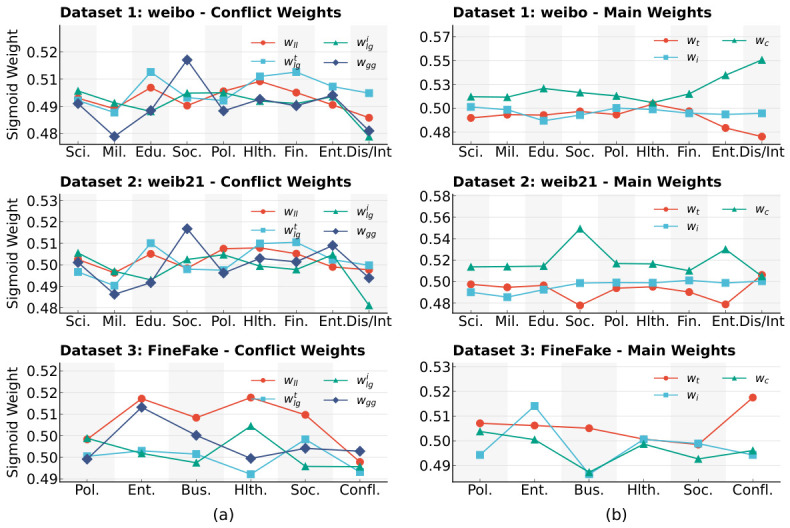
Line chart of (**a**) domain-adaptive multi-granularity conflict and (**b**) multi-channel feature fusion weights on the Weibo, Weibo21, and FineFake datasets.

To further validate the effectiveness of the domain-adaptive fusion mechanism, we analyze the dynamically learned routing weights of the model on Weibo, Weibo21, and FineFake. We use sigmoid weights as a quantitative measure of feature dependency across domains. The results are shown in Figure 8. Figure 8a and Figure 8b show the differences in sigmoid weights for conflicting patterns and multi-channel feature fusion, respectively. The sigmoid outputs are independent gate values rather than a normalized distribution. Although most values are close to 0.5, the relative ordering across features reliably reflects the model’s dependency strength.

In the integration of conflict modes and multi-channel features, the sigmoid weights across different channels vary with the domain, indicating that the model can adaptively adjust its reliance on each feature based on the domain attributes of the input. Specifically, in the Weibo and Weibo21 datasets, domains exhibit similar modulation patterns: the social and entertainment domains show relatively higher reliance on global semantic conflicts; the science, military, and political domains exhibit slightly stronger weights for semantic conflicts between image-local and text-global features; the health, education, finance, and disaster domains tend to display an increased dependency on semantic conflicts between text-local and image-global features. Regarding multi-channel features, conflict-fusion features exhibit slightly higher weights. In the FineFake dataset, the sigmoid weights for fine-grained local–local conflict features show relatively higher activation levels across most domains. However, in the conflict and politics domains, the sigmoid weights for the four conflict types are relatively close, primarily due to the official and rigorous writing styles that characterize these two domains. Additionally, among the multi-channel features, text features have relatively higher sigmoid weights in the politics, business, and conflict domains, while image features and conflict features receive slightly lower weights.

#### 4.4.2. Parameter Analysis

**Figure 9:**
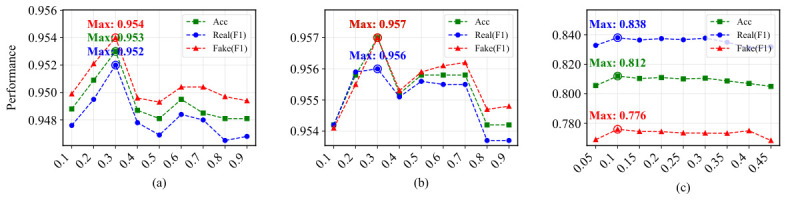
The performances of GC^2^MFND with different values of α on (**a**) Weibo, (**b**) Weibo21, and (**c**) FineFake.

**Figure 10:**
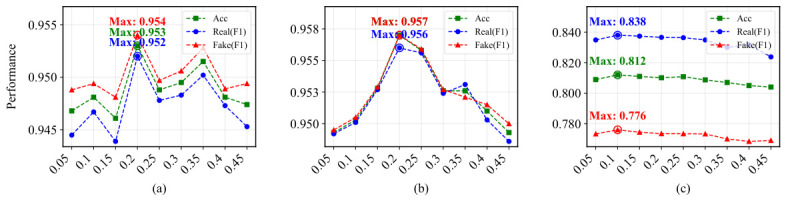
The performances of GC^2^MFND with different values of β on (**a**) Weibo, (**b**) Weibo21, and (**c**) FineFake.

**Figure 11:**
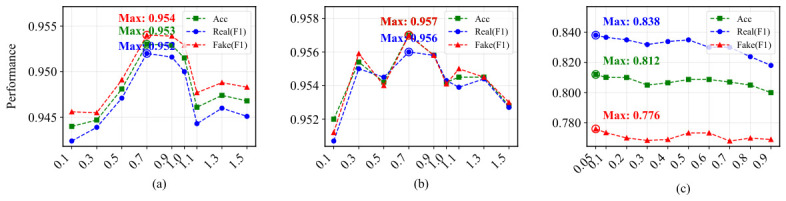
The performances of GC^2^MFND with different values of γ on (**a**) Weibo, (**b**) Weibo21, and (**c**) FineFake.

**Figure 12:**
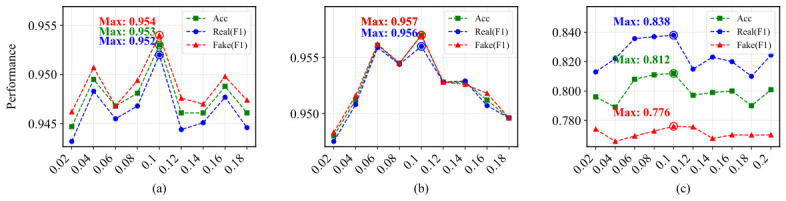
The performances of GC^2^MFND with different values of τ on (**a**) Weibo, (**b**) Weibo21, and (**c**) FineFake.

We analyze the sensitivity of the method to different values of the parameters α, β, γ, and τ on the Weibo, Weibo21, and FineFake datasets. Figure 9, Figure 10, Figure 11 and Figure 12 present the experimental results for these four key parameters. Overall, the Chinese datasets are more sensitive to parameter variations. The English dataset exhibits lower sensitivity. For the conflict feature loss parameter α, the model peaks at 0.3 on the Chinese datasets. On the English dataset, favorable results are observed within [0.08,0.12], with 0.1 yielding a better outcome. For the correction loss parameter β, GC^2^MFND works well at 0.2 on the Chinese datasets and at 0.1 on the English dataset.

Regarding the contrastive loss parameter γ, the Weibo and Weibo21 datasets perform better at 0.7. The FineFake dataset achieves better performance within [0.04,0.06], with 0.05 giving higher accuracy. For the contrastive loss temperature parameter τ, GC^2^MFND shows consistent performance at 0.1 across all three datasets, which is a robust choice. Furthermore, the performance drop across all three datasets under the same parameter settings did not exceed 0.009, and the performance remained higher than the respective baselines. This indicates that the model is stable. Based on these findings, we set the parameters for the Chinese datasets as α=0.3, β=0.2, γ=0.7, τ=0.1. For the English dataset, we use α=0.1, β=0.1, γ=0.05, τ=0.1.

#### 4.4.3. Computational Cost Analysis

To comprehensively evaluate the computational efficiency of GC^2^MFND, we compare the average single-round training time, testing time, inference time, GPU memory consumption, and number of parameters across various models on Weibo, Weibo21, and FineFake under a unified experimental setting. We select the two best-performing baselines (MMDFND and DAMMFND) for a fair comparison. Since all models share the same pre-trained feature extractor, differences in time and parameters arise solely from their respective downstream network designs.

Table 7 presents the computational overhead metrics. GC^2^MFND demonstrates a clear advantage in parameter efficiency, with a trainable parameter count of only 6.82 million, considerably lower than the two baselines. This advantage stems from architectural differences: MMDFND and DAMMFND employ multiple expert networks or domain-aware Transformer decoders, leading to a large parameter count; in contrast, GC^2^MFND uses only lightweight operators, gated networks, multi-scale adapters, low-dimensional domain embeddings, conflict extraction and calibration modules based on linear mappings and attention, as well as an MLP classifier.

On Weibo and Weibo21, GC^2^MFND demonstrates competitive training efficiency, with a single-round training time around 45.27 s and 43.03 s, respectively. This is approximately 25% faster than DAMMFND. Its testing time is approximately 50% faster than that of MMDFND and comparable to that of DAMMFND. On the larger-scale FineFake dataset, DAMMFND achieves the best training efficiency because it relies solely on discrete-domain routing. In contrast, GC^2^MFND incorporates fine-grained cross-modal interaction and attention calibration, which increases the computational complexity of matrix operations as the dataset size grows, thereby resulting in longer training times.

**Figure 13:**
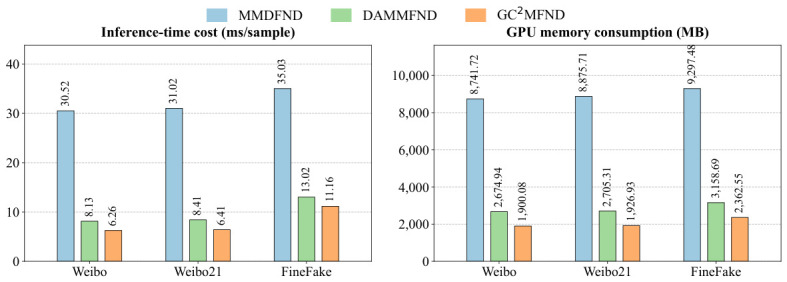
Comparison of inference time and GPU memory consumption across different methods.

However, in practical deployment, the timeliness of online inference is of greater importance. As shown in Figure 13, GC^2^MFND exhibits the lowest inference time and GPU memory consumption across the three datasets, indicating that its high accuracy does not come at the expense of real-time responsiveness. With a small number of parameters and efficient online inference, it can meet the need for timely detection and blocking of fake news in social media environments without incurring high computational costs.

#### 4.4.4. Case Study and Error Analysis

**Figure 14:**
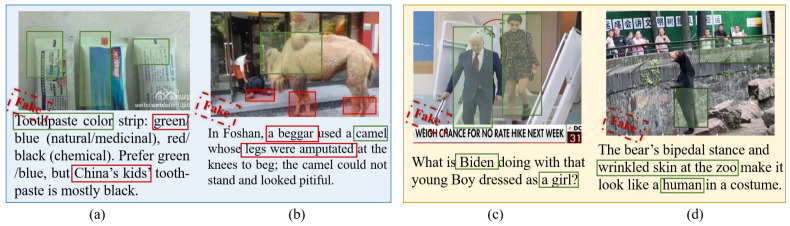
Examples from the Weibo21 and FineFake datasets. Figure (**a**) shows a local conflict between the text “toothpaste” and the image illustrating the hazards of children’s toothpaste; Figure (**b**) presents a global conflict between the text “camels begging” and the image “a camel being led by a person” as well as a local conflict between the text “amputated limbs” and the image “a camel with normal limbs”; Figure (**c**) indicates consistency between the image and the textual references to “Biden” and “girl”; Figure (**d**) reveals high consistency between the text and image in elements such as “bear,” “bipedal stance,” “wrinkled skin,” and “human”.

To evaluate the proposed model, we qualitatively analyze correctly and incorrectly predicted cases, as shown in Figure 14. Case (a) is a correctly identified fake news item. Although the text mentioning “Toothpaste” matches certain colors and visual elements, a subtle local conflict exists regarding children’s toothpaste and its hazards. Case (b) is also correctly identified: the phrase “camels begging” globally conflicts with an image showing “a camel being led by a person.” Additionally, the phrase “amputated limbs” locally conflicts with an image of “a camel with normal limbs.” In contrast, Case (c) is a misclassified fake news item. Although there is no explicit visual–textual conflict between the image of a “girl” and the text mentioning “Biden”, external knowledge confirms that the child is Biden’s granddaughter, exposing the caption’s false claim about a “young Boy dressed as a girl”. Case (d) is another misclassified example. The text and the image are highly consistent regarding elements such as “bear,” “bipedal stance,” “wrinkled skin,” and “human,” which misleads the model into an incorrect prediction. However, incorporating external knowledge—that sun bears have loose, clothing-like wrinkles and an eerily human-like posture—is required to correctly identify it as fake.

Based on the case studies above, the multi-granularity conflict features extracted by GC^2^MFND can effectively capture cross-modal inconsistencies between textual and visual content, thereby facilitating the detection of subtle fake news instances. However, as shown in Cases (c) and (d), when textual and visual information is highly consistent, conflict signals alone may be insufficient to reveal that the news is false, and external factual knowledge is often needed. Therefore, integrating external knowledge may further improve fake news detection performance in complex scenarios.

## 5. Conclusions

This study proposes a multimodal fake news detection framework (GC^2^MFND) designed for multi-domain scenarios. To address the issue of domain heterogeneity, the framework leverages domain embeddings to achieve a deep decoupling and integration of domain knowledge with cross-modal conflict mining, effectively mitigating feature semantic drift. Specifically, the framework first utilizes a domain-aware multi-granularity module to accurately extract text–image conflict signals. Subsequently, it employs a domain-guided feature calibration and redundancy reduction strategy to filter out redundant noise. Finally, a domain-adaptive dynamic aggregation and multi-view integration module is utilized to perform collaborative decision-making. Extensive experiments on three Chinese and English datasets—Weibo, Weibo21, and FineFake—demonstrate that GC^2^MFND achieves consistent improvements over existing multi-domain baseline methods. Ablation studies, mechanism analyses, and case studies further confirm that the conflict extraction and feature correction operations effectively enhance the discriminative power of features, while the dynamic aggregation strategy improves the model’s domain adaptability across complex topics.

Although GC^2^MFND achieves better performance in multi-domain fake news detection, this study still has certain limitations. First, the model relies on explicit domain labels, but many social media news items lack predefined domain classifications, resulting in high manual annotation costs. Furthermore, the model’s robustness in handling unseen scenarios requires further validation. Additionally, relying solely on news content without incorporating external knowledge makes the model prone to miss highly deceptive fake news. Future work will explore cross-domain transfer learning to reduce reliance on target domain labels, incorporate external knowledge bases to enhance detection capabilities for unknown domains and sophisticated samples, and adopt weakly supervised or unsupervised methods to lower manual annotation costs.

## Figures and Tables

**Table 1:** Notations and definitions used in this paper.

Notation	Definition	Notation	Definition
$T_{\mathrm{local}},I_{\mathrm{local}}$	Local Image/Text Features	$T_{\mathrm{local}}^{\mathrm{mod}},I_{\mathrm{local}}^{\mathrm{mod}}$	Modulated Local Image/Text Features
$T_{\mathrm{global}},I_{\mathrm{global}}$	Global Image/Text Features	${\hat{T}}_{\mathrm{local}},{\hat{I}}_{\mathrm{local}}$	Purified features without redundancy
${\tilde{T}}_{\mathrm{local}},{\tilde{I}}_{\mathrm{local}}$	Enhanced Local Image/Text Features	$T_{\mathrm{calib}},I_{\mathrm{calib}}$	Calibrated Image/Text Features
${\tilde{T}}_{\mathrm{global}},{\tilde{I}}_{\mathrm{global}}$	Enhanced global Image/Text Features	$s^{m},sh^{m}$	Dynamic scaling factor and offset
$F_{ll},F_{lg}^{t},F_{lg}^{i},F_{gg}$	Multi-granularity conflict features	$R_{t\to i},R_{i\to t}$	Cross-modal redundant features
$w_{ll},w_{lg}^{t},w_{lg}^{i},w_{gg}$	Conflict feature fusion weights	$w_{t},w_{i},w_{c}$	Multimodal feature aggregation weights
$e_{d}$	Domain Embedding	${\mathrm{BCE}}_{\mathrm{smooth}}$	Smoothed BCE loss
$F_{C}$	Domain-Aware Conflict Features	$L_{\mathrm{final}}$	Final discriminative loss
$F_{m}$	Fused Multimodal Features	$L_{\mathrm{total}}$	Total loss
$F_{f}$	Final Discriminative Features	$L_{t}$	Text feature classification loss
$F_{\mathrm{seq}}\left(\cdot \right)$	Local Conflict Extraction Operator	$L_{i}$	Image feature classification loss
H(·)	Global Conflict Extraction Operator	$L_{\mathrm{DSC}}$	Domain-Aware Soft-Weighted Contrastive Loss
Res-FiLM	Residual Feature-wise Linear Modulation	DGR	Domain-guided Gated Redundancy Removal

**Table 2:** Statistics of the datasets used in our experiments.

Datasets	Fake News	Real News	All
Weibo	4783	4745	9528
Weibo21	4487	4640	9127
FineFake	6402	10,507	16,909

**Table 3:** Comparison between GC^2^MFND and the latest multi-domain fake news detection methods on Weibo, Weibo21 and FineFake.

Dataset	Method	Sci.	Mil/Con	Edu.	Soc.	Pol.	Hlth.	Fin.	Ent.	Dis/Int	All		
											F1	Acc	Auc
Weibo	MOSE *	0.793	0.738	0.834	0.912	0.764	0.859	0.791	0.844	0.883	0.890	0.890	0.954
	KATMF *	0.831	0.908	0.924	0.895	0.823	0.898	0.903	0.904	0.894	0.929	0.930	0.969
	MDFEND *	0.774	0.911	0.897	0.902	0.763	0.878	0.808	0.881	0.874	0.904	0.904	0.965
	M^3^DFEND *	0.792	0.903	0.923	0.912	0.765	0.863	0.899	0.899	0.876	0.928	0.928	0.969
	MMDFND *	0.824	0.911	0.941	0.939	0.735	0.913	0.917	0.917	0.888	0.934	0.934	0.972
	DAMMFND	**0.853**	0.911	0.956	0.943	0.822	0.939	**0.937**	**0.956**	**0.928**	0.942	0.942	0.982
	**GC^2^MFND**	**0.853**	**0.956**	**0.972**	**0.957**	**0.823**	**0.948**	0.917	0.940	0.889	**0.953**	**0.953**	**0.986**
Weibo21	MOSE *	0.850	0.885	0.881	0.872	0.880	0.917	0.867	0.891	0.867	0.893	0.894	0.954
	KATMF *	0.914	0.928	0.913	0.895	0.902	0.914	0.871	0.937	0.898	0.923	0.928	0.975
	MDFEND *	0.830	0.938	0.891	0.898	0.886	0.940	0.895	0.906	0.900	0.913	0.913	0.970
	M^3^DFEND *	0.829	0.950	0.899	0.908	0.882	**0.946**	0.900	0.931	0.889	0.921	0.921	0.975
	MMDFND *	0.937	0.953	0.852	**0.945**	0.965	0.920	0.884	0.959	0.919	0.939	0.939	0.977
	DAMMFND	0.932	0.948	0.931	0.916	0.978	0.919	0.937	0.970	0.944	0.945	0.945	0.983
	**GC^2^MFND**	**0.988**	**0.998**	**0.965**	0.928	**0.982**	0.936	**0.941**	**0.980**	**0.945**	**0.957**	**0.957**	**0.986**
FineFake	MOSE	-	0.691	-	0.788	0.732	0.786	0.777	0.831	-	0.773	0.775	0.851
	KATMF	-	0.662	-	0.778	0.740	0.813	0.803	0.824	-	0.776	0.778	0.861
	MDFEND	-	0.699	-	0.781	0.742	0.796	0.810	0.830	-	0.782	0.785	0.873
	M^3^DFEND	-	0.648	-	**0.813**	0.732	0.762	0.827	**0.857**	-	0.781	0.781	0.873
	MMDFND	-	0.694	-	0.779	0.752	0.803	0.840	0.832	-	0.784	0.789	0.875
	DAMMFND	-	**0.713**	-	0.807	0.769	0.827	0.833	0.833	-	0.795	0.798	0.882
	**GC^2^MFND**	-	0.693	-	0.808	**0.785**	**0.830**	**0.842**	0.788	-	**0.807**	**0.812**	**0.890**

Bold: best results, Underline: second best results.

**Table 4:** Comparison of the stability and *p*-values of GC^2^MFND with two strong baseline methods across three datasets.

Datasets	Method	Accuracy	Precision	Recall	F1
Weibo	MMDFND	92.96 ± 0.80	92.99 ± 0.80	92.97 ± 0.81	92.95 ± 0.80
	DAMMFND	93.27 ± 0.48	93.31 ± 0.48	93.25 ± 0.49	93.26 ± 0.48
	**GC^2^MFND**	**94.78 ± 0.26**	**94.78 ± 0.26**	**94.82 ± 0.26**	**94.78 ± 0.26**
	*p*-value	$2.842\times 10^{-5}$	$3.375\times 10^{-5}$	$2.741\times 10^{-5}$	$2.693\times 10^{-5}$
		$5.761\times 10^{-7}$	$7.672\times 10^{-7}$	$4.871\times 10^{-7}$	$5.350\times 10^{-7}$
Weibo21	MMDFND	93.19 ± 0.84	93.21 ± 0.84	93.19 ± 0.85	93.19 ± 0.85
	DAMMFND	94.02 ± 0.87	94.03 ± 0.87	94.02 ± 0.87	94.02 ± 0.87
	**GC^2^MFND**	**95.07 ± 0.38**	**95.09 ± 0.38**	**95.08 ± 0.39**	**95.07 ± 0.38**
	*p*-value	$2.943\times 10^{-5}$	$2.943\times 10^{-5}$	$2.885\times 10^{-5}$	$3.112\times 10^{-5}$
		$2.023\times 10^{-4}$	$1.745\times 10^{-4}$	$1.642\times 10^{-4}$	$2.023\times 10^{-4}$
FineFake	MMDFND	75.29 ± 4.02	75.15 ± 4.15	74.62 ± 4.18	74.73 ± 4.22
	DAMMFND	79.32 ± 0.94	79.27 ± 1.04	78.79 ± 0.82	78.92 ± 0.88
	**GC^2^MFND**	**80.77 ± 0.69**	**80.63 ± 0.79**	**80.41 ± 0.65**	**80.47 ± 0.66**
	*p*-value	$1.902\times 10^{-3}$	$2.411\times 10^{-3}$	$1.672\times 10^{-3}$	$1.943\times 10^{-3}$
		$1.173\times 10^{-3}$	$4.341\times 10^{-3}$	$1.391\times 10^{-4}$	$3.834\times 10^{-4}$

Bold: best results. Note: The *p*-values are listed in order as MMDFND and DAMMFND.

**Table 5:** Performance comparison across different datasets.

Datasets	Method	Accuracy	F1	
			Fake News	Real News
Weibo	EANN	0.827	0.829	0.825
	SpotFake	0.892	0.932	0.739
	CAFE	0.840	0.842	0.837
	BMR	0.918	0.914	0.904
	MTS	0.925	0.926	0.924
	MIAN	0.936	0.935	0.937
	**GC^2^MFND**	**0.953**	**0.954**	**0.952**
Weibo21	EANN	0.870	0.862	0.875
	SpotFake	0.851	0.828	0.866
	CAFE	0.882	0.885	0.876
	BMR	0.929	0.927	0.925
	MTS	0.928	0.929	0.927
	MIAN	0.938	0.936	0.939
	**GC^2^MFND**	**0.957**	**0.956**	**0.957**
FineFake	EANN	0.785	0.770	0.792
	SpotFake	0.779	0.760	0.791
	CAFE	0.783	0.769	0.798
	BMR	0.783	0.729	0.819
	MIAN	0.784	0.759	0.804
	MTS	0.788	0.758	0.811
	**GC^2^MFND**	**0.812**	0.776	**0.838**

Bold: best results.

**Table 6:** Ablation study results on different datasets.

Datasets	Method	Accuracy	F1	
			Fake News	Real News
Weibo	**GC^2^MFND**	**0.953**	**0.954**	**0.952**
	-w/o_Conflict	0.944	0.944	0.943
	-w/o_Calib	0.939	0.941	0.938
	-w/o_Domain	0.941	0.942	0.939
	-w/o_Loss	0.948	0.949	0.947
	-w/o_Smooth	0.941	0.942	0.939
Weibo21	**GC^2^MFND**	**0.957**	**0.956**	**0.957**
	-w/o_Conflict	0.949	0.950	0.949
	-w/o_Calib	0.941	0.942	0.940
	-w/o_Domain	0.944	0.944	0.944
	-w/o_Loss	0.941	0.942	0.939
	-w/o_Smooth	0.942	0.943	0.940
FineFake	**GC^2^MFND**	**0.812**	**0.776**	**0.838**
	-w/o_Conflict	0.808	0.769	0.836
	-w/o_Calib	0.808	0.775	0.832
	-w/o_Domain	0.806	0.771	0.832
	-w/o_Loss	0.801	0.758	0.831
	-w/o_Smooth	0.804	0.779	0.824

Bold: best results.

**Table 7:** Computational efficiency comparison of different methods.

Method	Training Time (s)			Testing Time (s)			Parameters (M)
	Weibo	Weibo21	FineFake	Weibo	Weibo21	FineFake	
MMDFND	302.95	275.44	744.87	35.59	27.10	249.86	347.82
DAMMFND	61.93	57.61	**164.96**	**15.97**	13.98	**71.44**	76.62
**GC^2^MFND**	**45.27**	**43.03**	360.43	18.13	**13.96**	120.31	6.82

Bold: best results.

## Data Availability

The three datasets used in this study are all publicly available and can be obtained from the relevant cited articles. The source code for GC^2^MFND can be found at https://github.com/ZMingYue-Z/GC2MFND (accessed on 20 April 2026).
